# Harnessing the Transcriptional Signatures of CAR-T-Cells and Leukemia/Lymphoma Using Single-Cell Sequencing Technologies

**DOI:** 10.3390/ijms25042416

**Published:** 2024-02-19

**Authors:** Yu-Mei Liao, Shih-Hsien Hsu, Shyh-Shin Chiou

**Affiliations:** 1 Division of Hematology-Oncology, Department of Pediatrics, Kaohsiung Medical University Hospital, Kaohsiung Medical University, Kaohsiung 807, Taiwan; p920271@gmail.com; 2 Graduate Institute of Medicine, College of Medicine, Kaohsiung Medical University, Kaohsiung 807, Taiwan; 3 Department of Medical Research, Kaohsiung Medical University Hospital, Kaohsiung Medical University, Kaohsiung 807, Taiwan; 4 Research Center for Environmental Medicine, Kaohsiung Medical University, Kaohsiung 807, Taiwan; 5 Center of Applied Genomics, Kaohsiung Medical University, Kaohsiung 807, Taiwan; 6 Graduate Institute of Clinical Medicine, College of Medicine, Kaohsiung Medical University, Kaohsiung 807, Taiwan

**Keywords:** single-cell sequencing technologies, CAR-T therapy, tumor microenvironment, transcriptional complexity

## Abstract

Chimeric antigen receptor (CAR)-T-cell therapy has greatly improved outcomes for patients with relapsed or refractory hematological malignancies. However, challenges such as treatment resistance, relapse, and severe toxicity still hinder its widespread clinical application. Traditional transcriptome analysis has provided limited insights into the complex transcriptional landscape of both leukemia cells and engineered CAR-T-cells, as well as their interactions within the tumor microenvironment. However, with the advent of single-cell sequencing techniques, a paradigm shift has occurred, providing robust tools to unravel the complexities of these factors. These techniques enable an unbiased analysis of cellular heterogeneity and molecular patterns. These insights are invaluable for precise receptor design, guiding gene-based T-cell modification, and optimizing manufacturing conditions. Consequently, this review utilizes modern single-cell sequencing techniques to clarify the transcriptional intricacies of leukemia cells and CAR-Ts. The aim of this manuscript is to discuss the potential mechanisms that contribute to the clinical failures of CAR-T immunotherapy. We examine the biological characteristics of CAR-Ts, the mechanisms that govern clinical responses, and the intricacies of adverse events. By exploring these aspects, we hope to gain a deeper understanding of CAR-T therapy, which will ultimately lead to improved clinical outcomes and broader therapeutic applications.

## 1. Introduction

Chimeric antigen receptor (CAR)-T-cell therapy has emerged as a crucial component of immunotherapy for cancer, particularly in the treatment of hematological malignancies [1,2,3]. A CAR is an engineered receptor composed of four main parts: the extracellular, hinge, transmembrane, and intracellular signal transduction domains. The extracellular domain comprises a single chain fragment variable (scFv) that can recognize and bind to the specific antigen on the malignant cells. The hinge, transmembrane, and signal transduction domains of CAR-T-cells consist of a dimer of homologous or heterologous combinations of CD3, CD8, and CD28. These elements regulate receptor expression and signaling thresholds subsequently mediating optimal cellular activation [4,5]. The signal transduction domains of CAR-T-cells can control the input efficiency of CAR signals, facilitating the cytokine production function, and enhancing the cytotoxicity of CAR-T-cells [6]. The heterogeneity of CAR-T-cell products is influenced by the CAR structure [7,8,9], T-cell subtype [10], and product manufacturing process [11], consequently impacting the efficacy and safety of CAR-T-cell therapy. Various combinations of molecular modules of the CAR, such as the selection of the costimulatory domain [9] and the immunogenicity of the single-chain variable fragment (scFv) [7,8], result in different effects on the phenotype and function of CAR-T-cells.

CAR-T-cells exhibiting less-differentiated naïve and early memory features have been found to correlate with a higher rate of durable clinical remission [12,13]. Conversely, exhausted T-cells characterized by increased expression of inhibitory immune checkpoint receptors are associated with poorer clinical outcomes [14]. Furthermore, variations in CAR-T-cell lineage clones that display distinct patterns of clonal kinetics contribute to the variety of the postinfusion CAR-T-cell pool. Notably, the manufacturing process introduces heterogeneity at each step, impacting the final composition of the CAR-T-cell product [11].

The U.S. Food and Drug Administration (FDA) has approved six CAR-T-cell products (Table 1) since 2017 [15,16,17,18,19]. However, obstacles such as treatment resistance to CAR-T, relapse after CAR-T therapy, and serious adverse effects hinder their clinical applications [20,21]. Additionally, CAR-T-cell therapy demonstrates limited efficacy in treating solid tumors compared to its effectiveness in hematological malignancies [22,23,24,25,26]. This limitation may be attributed to inadequate tumor infiltration of CAR-T-cells, the lack of stable expression of tumor-specific antigens, and the highly immunosuppressive tumor microenvironment (TME) [27,28,29,30,31].

After the completion of the Human Genome Project, the structure and functions of the genome have been extensively explored. The development of cancer is theoretically an accumulation of genetic and epigenetic changes, including the loss of insensitivity to regulatory factors, uncontrolled growth and cell division, and the loss of apoptosis within the cell. The genetic alterations and global changes within cancer cells can be dissected at genomic, transcriptomic, and proteomic levels. Each of these aspects has its unique advantages and limitations [34].

The genomic study level is the first aspect that has been extensively explored, with rapid progress made after the sequencing of the human genome [35]. Specific mutations responsible for malignant transformation and hereditary cancer syndromes have been identified [36]. However, genomic studies have their limitations. First, only a small percentage of the human genome is expressed down to the protein level. Secondly, gene expression is a complex process regulated by various mechanisms, such as DNA methylation [37], DNA-binding proteins [38], or small interfering RNA (siRNA) [39]. Therefore, the genomic level alone cannot fully represent the cancer phenotype, necessitating the exploration of other approaches to address this crucial issue.

The proteomic approach is much closer to unraveling the molecular mechanisms determining a cell’s phenotype compared to the genomic approach [40]. However, the proteomic approach, such as protein microarrays, also has its limitations, particularly regarding the diverse physical and chemical properties of monoclonal antibodies. Therefore, over the past decades, transcriptome profiling has emerged as one of the most powerful tools in the field of oncology. The transcriptome encompasses the sum of all RNA transcripts in an individual cell or bulky tumor cells. It includes protein-coding RNAs (pcRNAs), commonly known as messenger RNAs (mRNAs), and noncoding RNAs (ncRNAs). Each of these RNAs plays a distinct role in the cell and responds variably to environmental stimuli [41,42].

The early stages of transcriptome annotations began with cDNA libraries published in the 1980s. Two biological techniques are commonly used to study the transcriptome: DNA microarray, a hybridization-based technique, and RNA-seq, a sequence-based approach [34]. Subsequently, the advent of high-throughput technology led to faster and more efficient ways of obtaining data about the transcriptome. Nowadays, next-generation sequencing has become the preferred method and has dominated the field of dominant transcriptomics since the 2010s.

Data obtained from the transcriptome can be utilized for research on cellular differentiation process, carcinogenesis, transcription regulation, and biomarker discovery in the field of oncology. Conventional next-generation sequencing can detect the transcriptomic information of cell populations, such as a large number of cells, animal and plant tissues, or even an organism. However, bulky next-generation sequencing has its limitation in dissecting cell heterogeneity within the bulky tumors. 

**Figure 1:**
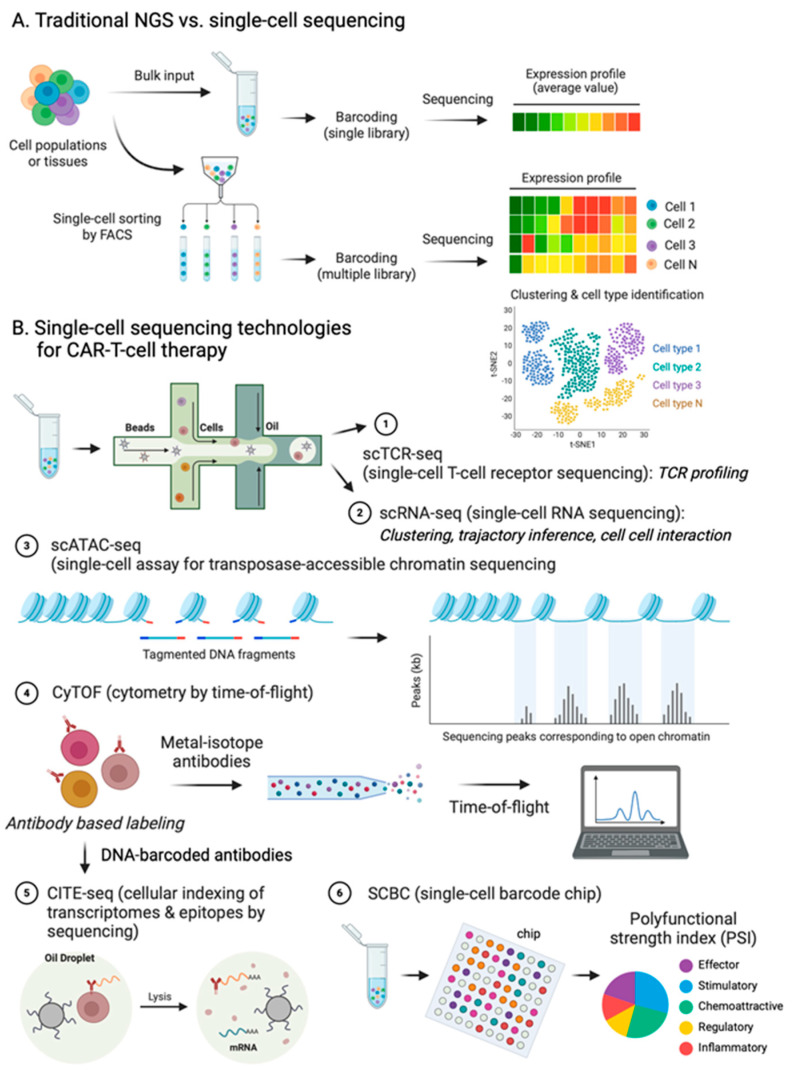
*The differences between NGS sequencing protocols and principles of the main single-cell technologies* (**A**) Traditional next-generation sequencing (NGS) examines the genetic material of a group of cells, such as a cell culture, tissue, organ, or entire organism. The outcome is the representation of the “average genome” within that cell population. Conversely, single-cell sequencing scrutinizes the genetic content of individual cells within the same cell population. (**B**) Single-cell sequencing analysis technologies for CAR-T-cell therapy. Schematic representation of single-cell multiomics analysis in CAR-T-cell therapy. ① scTCR-seq concurrently profiles the V(D)J sequence of TCR and gene expression patterns. ② scRNA-seq captures the entire cellular transcriptome. ③ scATAC-seq identifies open chromatin regions by utilizing adapters with Tn5 transposase to map transcription factor binding sites. ④ CyTOF enables high-dimensional, multiparametric protein detection using metal-isotype antibodies, inductively coupled plasma ionization, and time-of-flight detection. ⑤ CITE-seq facilitates the simultaneous evaluation of the transcriptome and surface or intracellular protein expression in individual cells, immunostained with oligonucleotide-coupled antibodies. ⑥ SCBC measures multiple secreted proteins using arrays of microchambers decorated with ordered arrays of antibodies against target proteins.

While traditional techniques allow for the quantitative profiling of genomic, transcriptomic, and proteomic signatures in bulk tumors, the advent of single-cell sequencing has catalyzed significant advancements in identifying innovative biomarkers, novel cellular phenotypes, and new therapeutic targets that remain undetectable through bulk-sequencing approaches [43,44,45] (Figure 1A). Despite a limited sensitivity to detect low-abundance RNAs and “technical noise” due to the low amount of input material [46,47], single-cell sequencing, with its high resolution, serves as an invaluable tool for studying the properties of immune cells. This includes exploring diverse developmental lineages, antigen specificity, phenotypic plasticity, cellular transitions, interactions, cellular communications, and adaptability to various microenvironments [45,48,49,50,51,52,53,54,55,56].

Various modern single-cell sequencing technologies have been employed in CAR-T-cell research, including scRNA-seq, single-cell T-cell receptor sequencing (scTCR-seq), single-cell assay for transposase-accessible chromatin sequencing (scATAC-seq), cytometry by time-of-flight (CyTOF), cellular indexing of transcriptomes and epitopes by sequencing (CITE-seq), and single-cell multiplexed secretome proteomics (Figure 1B).

Of these tools, scRNA-seq currently stands out as the most widely used single-cell sequencing technology. The droplet-based platform (10x Genomics BD) is the predominant scRNA-seq platform in use. Using a microfluidic chamber, the droplet-based method separates individual cells into an oil-based microdroplet. Each droplet contains gel microbeads with mRNA-capturing primers in conjunction with a unique molecular barcode alongside an enzyme/reagent mix, which is required for cell lysis and reverse transcription. After reverse transcription and cDNA amplification, a barcoded sequencing library is generated. Subsequently, the samples undergo processing for sequencing and subsequent analyses [57,58]. In scTCR-seq, the 5′-end transcript is sequenced, allowing for the simultaneous detection of the V(D)J sequence and transcript of a single cell. Consequently, scTCR-seq proves particularly useful in characterizing the clonotypic diversity of the T-cell population, and dissecting T-cell functional phenotypes, such as activation, memory, and exhaustion T-cells [59,60,61]. scATAC-seq is primarily designed for measuring genomewide open chromatin regions to identify activated genes, promoters, and enhancers and subsequently postulate the transcription factors (TFs) to which cis-elements are bound [62]. This is achieved by inserting sequencing adapters into accessible regions of the genome using Tn5 transposase. As a result, scATAC-seq is mainly utilized for charactering the epigenetics of T-cell differentiation and exhaustion [63,64]. CyTOF is a combination of flow cytometry with mass spectrometry, enabling multiparameter analysis. Through conjugation with over 100 antibodies labeled with unique isotopically pure metals, CyTOF accurately detects intracellular and extracellular protein expression accurately at the single-cell level [65,66]. When combined with single-cell RNA-Seq, CyTOF becomes a powerful tool for characterizing the heterogeneity of rare T-cell subpopulations. CITE-seq exploits an oligonucleotide-barcoded antibody conjugated to the cell surface antigen to characterize cell populations based on surface protein markers and the transcriptome landscape [67]. This method allows for the simultaneous delineation of the immunophenotypic profile and transcriptome signature of T-cell. SCBC (single-cell barcode chip) utilizes concentrated microfabricated compartments with spatially barcoded capture sites for the highly multiplexed single-cell analysis of up to 42 secreted cytokines related to T-cell function, including effector, stimulatory, regulatory, and inflammatory molecules [68,69]. Given the significant heterogeneity in cytokine secretion observed in CAR-T-cells, the concept of polyfunctional CAR-T-cells and the polyfunctionality strength index (PSI) are employed to describe CAR-T-cell subsets capable of coproducing multiple cytokines at the single-cell level. This approach has been utilized to predict the clinical outcomes of patients [70,71,72,73].

The aforementioned single-cell methods play a crucial role in facilitating improved receptor design, guiding gene-based T-cell modification, and optimizing manufacturing conditions [64,74,75,76,77]. Furthermore, they contribute to the clinical prediction of therapeutic efficacy and toxicities, enabling a personalized treatment of CAR-T therapy [78,79]. Despite their significance, there have been relatively few reports reviewing the role of modern single-cell sequencing technologies in deciphering CAR-T-cell therapy [45,80,81,82,83].

In this review, our objectives are to address the potential mechanisms contributing to the clinical failures of CAR-T immunotherapy (Table 2). We delve into the biological characteristics of CAR-Ts, the mechanisms governing clinical responses, and the intricacies of adverse events. By exploring these aspects, we aim to gain a deeper understanding of CAR-T therapy, ultimately facilitating the optimal selection of target antigens, CAR-transgene integration, and manufacturing processes (Table 2), which collectively aim to improve clinical outcomes and broaden therapeutic applications.

## 2. Primary Resistance

**Figure 2:**
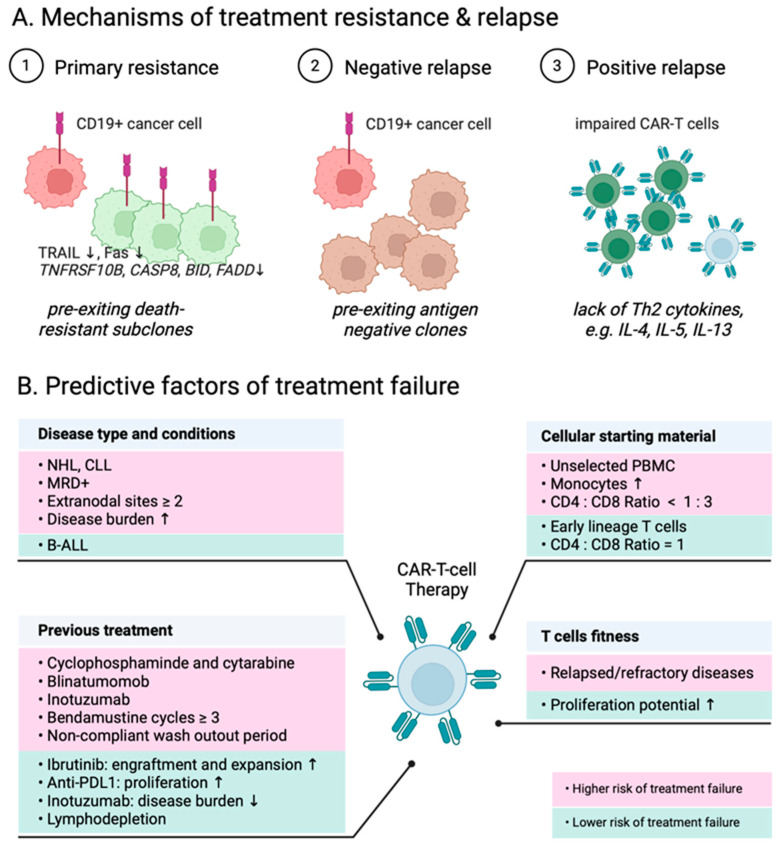
Major escape routes of leukemia or lymphomas and predictive factors for treatment failure. (**A**) The phenotypes of CAR-T-cells and leukemia/lymphoma cells are associated with ③ CD19-positive relapse, ② CD19-negative relapse, and ① primary treatment resistance. (**B**) Clinical and/or biological parameters that have already been identified may enhance the ability to predict the success of manufacturing and/or the clinical response to CAR-T-cell therapies. These parameters include aspects such as patient disease, prior treatments, characteristics of the cellular starting material, and the fitness of T-cells.

Primary resistance to CAR-T treatment is defined as CD19+ progressive disease during CD19 CAR-T treatment, with the underlying mechanisms remaining elusive. Several intrinsic mechanisms in leukemia have been identified (Table 3), including the loss of expression of death receptor signaling pathways such as TNF-related apoptosis-inducing ligand (TRAIL) and Fas pathway functions [95,110]. Notably, leukemia cells with a reduced expression of death receptor genes (Fas associated via death domain (FADD), BH3 interacting domain death agonist (BID), caspase 8 (CASP8), TNF receptor superfamily member 10b (TNFRSF10B)) have been associated with patients showing no response [95] (Figure 2A). Additionally, a stem cell epigenome in leukemia cells exhibiting myeloid and stem cell-like features is also linked to primary resistance to CD19 CAR-T treatment [111]. Sworder et al. reported alterations in multiple classes of genes in DLBCL cells, including those related to B-cell identity (paired box 5(PAX5) and interferon regulatory factor 8 (IRF8)), immune checkpoints (CD274), and the microenvironment transmembrane protein 30A (TMEM30A), were associated with resistance [112].

More significantly, novel single-cell technologies have played a crucial role in uncovering novel mechanisms involving T-cell dysfunction, such as high T-cell exhaustion markers and decreased CAR-T-cell expansion, which are factors associated with primary resistance disease [79] (Table 2). Deng et al. utilized a whole-transcriptome scRNA-seq analysis and found that exhausted CD4+ and CD8+ T cells were enriched within the product of patients with a partial response or progressive disease. In their analysis, the gene lymphocyte activation 3 (LAG-3) and T-cell immunoreceptor with Ig and ITIM domains (TIGIT) genes, as well as the basic leucine zipper ATF-like transcription factor (BATF) and inhibitor of DNA binding 2 (ID2), were highly expressed in those exhausted T-cells [79]. Jackson et al., utilizing scRNA-seq, also identified the upregulation of the exhaustion marker TIGIT in CAR-T-cells associated with a lower response in relapsed or refractory B-cell lymphoma patients [93]. CAR Treg cells have also been implicated in primary resistance [78,94]. Haradhvala et al., using scRNA-seq analysis, discovered that an increasing number of CAR Treg cells correlated with the nonresponse rate [94]. Good et al., utilizing CyTOF combined with single-cell RNA-seq, found that higher numbers of CD4+ HELIOS+ CAR-T-cells with a Treg cell phenotype were associated with disease progression [78].

## 3. Relapse

### 3.1. CD19Negative Relapse

A common reason for relapse after CAR-T-cell therapy is the loss of target antigen or loss of antigen expression [115] (Figure 2A). Possible mechanisms include the pre-exiting of antigen negative clones, such as CD34+CD19-CD22+ B-cell progenitors [97,113], gene mutation [116], splicing events [117], the transcriptional plasticity of leukemia cell [114], a reversible antigen loss through trogocytosis [118], the instant introduction of the CAR-T gene to the leukemia cell [119], and lineage switch-induced antigen loss [120].

On the other hand, factors associated with CAR-T-cells may also contribute to therapeutic failure [55,68,69,70,71,72,73,74,75,76,77,78,79,121,122,123,124]. It has been proposed that CD19-negative relapse is related to immune escape. Fraietta et al. reported that CAR-T-cells from nonresponders upregulated programs involved in effector differentiation, glycolysis, exhaustion, and apoptosis in CLL patients [12]. Moreover, programmed death-1 (PD-1)-mediated T-cell exhaustion is also involved in resistance to CAR-T-cell therapy [125,126,127].

### 3.2. CD19-Positive Relapse

The most common reason for relapse after CAR-T-cell therapy observed in many clinical trials is CD19-positive relapse [1]. The leukemia intrinsic factors contributing to CD19-positive relapse are still poorly understood. Additionally, CAR-T-associated factors, such as impaired CAR-T-cell activation, expansion, in vivo resistance, and poor antitumor potency [128,129], have also been found to be associated with CD19-positive relapse [129]. Bai et al., using an scRNA-seq analysis, discovered that a deficiency of TH2 function, as well as a lack of TH2 cytokine molecules such as IL-4, IL-5, and IL-13, were associated with CD19-positive relapse in patients [96] (Figure 2A). They also applied a CITE-seq analysis to identify novel subgroups of T-cells, revealing that early memory-like T-cell subsets T_SCM_ and T_CM_ were significantly decreased among patients with CD19--positive relapse [114].

### 3.3. Crosstalk between CAR-T-Cells and TME

The tumor microenvironment (TME) plays a critical role in cancer progression and relapse and is strongly associated with the failure of cancer immunotherapy. The immunosuppressive microenvironment includes immunosuppressive cells and cytokines. Cells such as tumor-associated macrophage, regulatory T-cells, myeloid-derived suppressor cells, and transforming growth factor β can inhibit the proliferation and effector functions of CAR-T-cells [130,131,132,133]. Deficiencies in chemokines also lead to impaired chemotactic abilities of CAR-T-cells [134]. Additionally, abnormal vasculature and cancer-associated fibroblasts make it challenging for CAR-T-cells to migrate to the tumor [135,136].

## 4. Adverse Events

**Figure 3:**
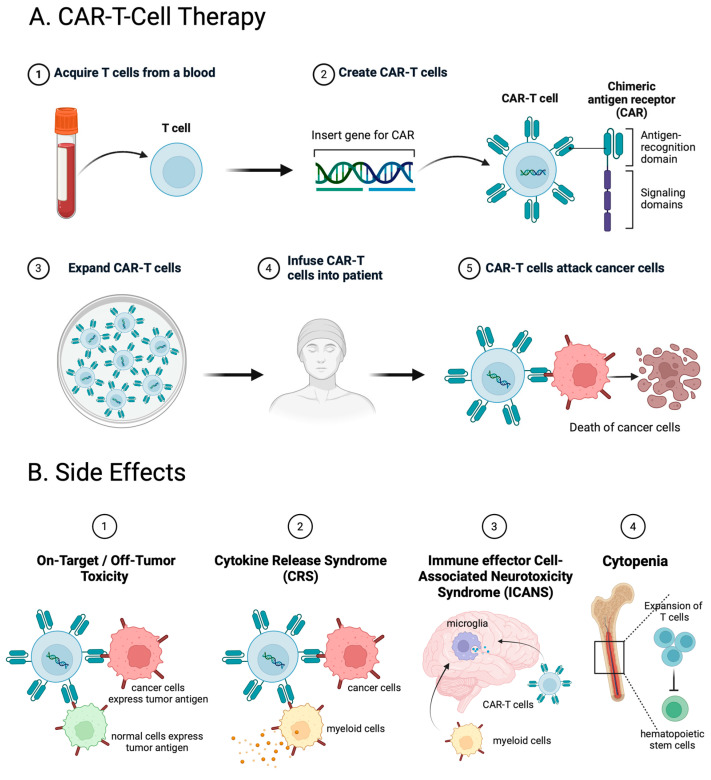
The basics of CAR-T-cells and side effects. (**A**) Flow diagram depicting the manufacturing process of CAR-T-cells. The process for producing autologous CAR-T-cells generally initiates with the leukapheresis of the patient’s immune cells. Subsequently, T-cells undergo activation and amplification using beads coated with antibodies. Following this, the CAR construct is integrated into the T-cells, commonly facilitated by viral or nonviral vectors. Lastly, CAR-T-cells undergo expansion to attain the necessary quantity before being infused into the patient, following rigorous quality control testing. (**B**) ① On-target, off-tumor toxicity occurs when the target antigen is expressed on both the tumor and healthy tissues in patients. ② IL-6, identified as crucial in the pathogenesis of CRS, exhibits specific and elevated expression in monocytes. ③ Targeting mural cells expressing CD19, which play a critical role in maintaining blood–brain barrier (BBB) integrity, may contribute to ICANS in CAR-T-cell therapy. ④ Cytopenias that arise following CAR-T infusion typically become evident early, within the first 30 days. They commonly persist for an extended period, ranging from 30 to 90 days, and in some cases, may either continue persisting or occur later, beyond 90 days. Association with the bone marrow infiltration of clonally expanded CD8 T-cells expressing IFNγ is one of the possible mechanisms.

The most common adverse events (AEs) of CAR-T cell therapy (Figure 3A) include cytokine release syndrome (CRS), immune effector cell-associated neurotoxicity syndrome (ICANS), cytopenia, infection, and on-target off-tumor effect [137,138] (Figure 3B). Single-cell analysis has played a significant role in deeper research into the underlying mechanisms of these AEs (Table 2).

### 4.1. CRS

Cytokine release syndrome (CRS) is the most common toxicity of CAR-T-cell therapy [139] (Figure 3B). Although reversible in the majority of cases, severe CRS can be fatal. Endothelial cell activation plays a crucial role during the initiation of CRS [140]. CRS is a systemic inflammatory response induced by endothelial cell dysfunction, the abnormal activation of macrophages, and the release of various proinflammatory cytokines such as IL-1, IL-6, and IL-8 [141,142]. Moreover, single-cell cytokine profiling has revealed that a higher product T-cell polyfunctionality strength index (PSI) is associated with grade ≥3 CRS [73]. Additionally, the combination of PSI with CAR-T-cell expansion or pretreatment serum IL-15 levels provides a more indicative measure of severe CRS [73].

Although is world-renowned that IL-6 is the main cytokine for CRS, the primary cellular source of IL-6 remains unknown. Norelli et al., utilizing scRNA-seq, sought to answer this important question. Their analysis of CD45+ leukocytes isolated from humanized mice that developed CRS after CAR-T-cell infusion confirmed that circulating monocytes were the sole cell population that specifically expressed high levels of IL-6 [142]. Furthermore, with the assistance of single-cell analyses, Deng et al. reported a positive association between exhausted CD4+ T-cells and a higher grade of CRS, while exhausted CD8+ T-cells had a negative association with a higher grade of CRS [79]. When comparing patients with grade 0-1 CRS to those with grade ≥2 CRS, it was found that the latter group had a higher PSI of CD4+ CAR-T-cells, especially the PSI of IL-8 and monocyte chemoattractant protein 1 (MCP-1) [72]. These cytokines are involved in the recruitment of neutrophils and monocytes/macrophages [143,144].

### 4.2. ICANS

Immune effector cell-associated neurotoxicity syndrome (ICANS) is another common adverse event associated with CAR-T-cell therapy (Figure 3B). The activation of endothelial cells in the brain, leading to inflammation and the disruption of the integrity of the blood–brain barrier (BBB), is the key factor in the development of ICANS [139,145]. With the assistance of single-cell analyses, Deng et al. identified a rare cell population with monocyte-like transcriptional features that was associated with high-grade ICANS [79]. 

On the other hand, Parker et al. discovered that CD19 is expressed during brain development, especially during the emergence of mural cell lineages. The mural cells surround the endothelium and are associated with the blood–brain barrier integrity. The expression of CD19 persists throughout adulthood in all brain regions as revealed by single-cell analysis. Consequently, brain mural cells expressing CD19 become off-tumor targets for CAR-T therapy [98]. Furthermore, Loeffler et al., utilizing scRNA-seq of 24 patients, found that the number of CAR+ cells, decreasing cell cycling activity, and the expression of cell cycle and exhaustion-related genes such as LAG3 and TIM3 were associated with a higher neurotoxicity [101].

In conclusion, single-cell sequencing technologies have the potential to identify biomarkers from CAR-T-cell products that predict an increased risk of severe ICANS. These biomarkers include ICANS-associated cells (IACs) with a monocyte-like transcriptional signature [79], an increased number of polyfunctional CAR-T-cells producing IL-17A [73], and low levels of CD4+Helios+ CAR-T-cells [78].

### 4.3. Cytopenia

Emerging data indicate the incidence of cytopenia induced by CAR-T therapy [146] (Figure 3B). Cytopenia of grade 3 or higher, lasting more than one month after CAR-T-cell infusion, occurs in approximately 20–40% of patients [147]. The mechanisms of delayed and persisting cytopenia after CAR-T-cell therapy are poorly understood. Various hypotheses have been proposed, including previous lines of therapies, greater age, poor bone-marrow reserve, the severity of CRS, and the roles of various inflammatory cytokines [147]. Singe-cell methods also help to further delineate the novel mechanisms. Rejeski et al. reported a case with dominant oligoclonal T-cell expansion in both CAR- and non-CAR-bearing T-cell populations. Those oligoclonal T-cells demonstrated an elevated expression of cell cycle-related genes and decreased expression of cell apoptosis, immune response, and inflammation-related genes [102]. Moreover, Paolo et al. discovered that prolonged cytopenia following CD19 CAR-T-cell therapy was linked to the bone marrow infiltration of clonally expanded IFN-γ-expressing CD8 T cells, using single-cell RNA-Seq and paired B-cell or T-cell receptor (BCR/TCR) sequencing [148]. In conclusion, single-cell technology provides previously undiscovered mechanisms of CAR-T-related hematologic toxicity.

### 4.4. Infection

Infection-related complications are also common among the CAR-T-treated patients with reports indicating that around 30% of patients experience a serious bacterial infection in the first 30 days after CAR-T treatment. Viral respiratory tract infections tend to occur during the late phase, while fungal infections and cytomegalovirus (CMV) reactivation are relatively uncommon [149]. Single-cell methods have also contributed to research in this field. Chen et al., using single-cell transcriptomics analysis, discovered that monocyte loss is one of the possible factors contributing to infections after CAR-T infusion [150] (Table 2).

## 5. CAR-T-Cell Behaviors Correlate with Clinical Therapeutic Response

Multiple factors are associated with CAR-T treatment responses, including the patient features, disease status, previous treatment, tumor characteristic, disease burden, cell starting materials, CAR-T product composition, and T-cell fitness [151,152] (Figure 2B). Here, we summarize recent single-cell studies that address the CAR-T-cell behaviors and their correlation to clinical therapeutic response.

### 5.1. The Dynamic and Kinetic Performance of CAR-T-Cells

**Figure 4:**
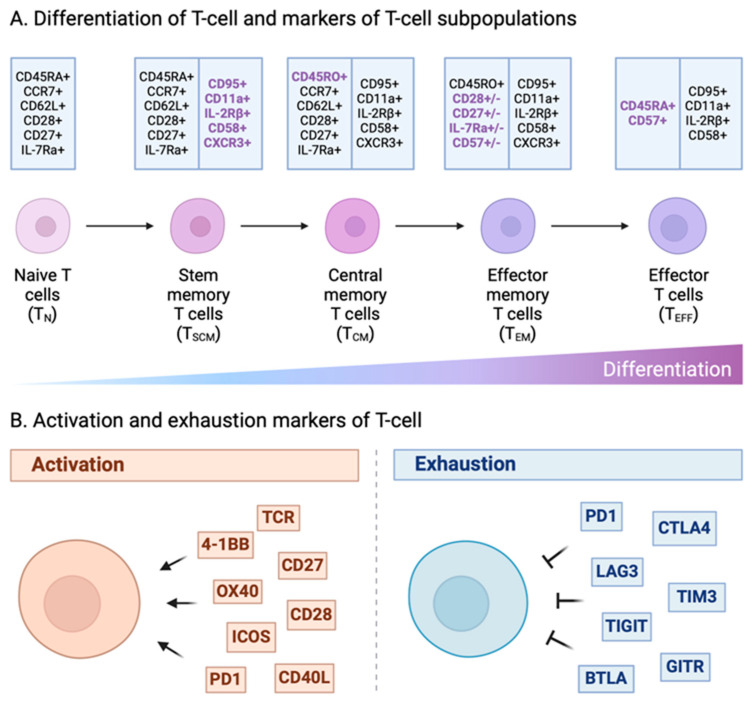
*A representation of T-cell differentiation along with markers identifying distinct T-cell subpopulations*. (A) Illustrative model depicting T-cell differentiation. Following activation, naïve T-cells undergo differentiation into diverse memory and effector cell types. The differentiation process is characterized by a decline in self-renewal capacity, multipotency, and proliferation potential. The expression of CD45R0, CCR7, CD28, and CD95 markers undergoes changes throughout T-cell differentiation, progressing from TN (naïve T-cell) to TEFF (terminal effector T-cell). A minimal set of canonical markers can effectively identify the five major T-cell subsets: TN, TSCM (stem-cell memory T-cell), TCM (central memory T-cell), TEM (effector memory T-cell), and TEFF. (B) Diagram illustrating T-cell exhaustion and markers associated with T-cell activation. Dysfunction of T-cells arises in situations of prolonged exposure to antigens, as observed in cancer contexts. This functional decline is termed T-cell exhaustion, encompassing the reduction in IL-2 production, diminished proliferative capacity, and compromised cytotoxic capabilities. This includes an impairment in the production of granzyme B, IFN-γ, and TNF-α. Concurrently with the loss of functionality, T-cells upregulate the expression of inhibitory receptors like PD1, LAG3, TIM3, CTLA4, and TIGIT. IFN-γ, interferon-γ; IL-2, interleukin 6; TNF-α, tumor necrosis factor α. The costimulatory domain is pivotal for CAR-T-cell persistence and efficacy. Notable examples of costimulatory molecules include CD28, ICOS, CD27, 4-1BB, OX40, and CD40L.

CAR-T-cell phenotypes undergo variations from the preinfusion product throughout the course of treatment. The heterogeneity in T-cell composition of CAR-T-cell products influences the differentiation of CAR-T-cells, leading to different cellular dynamics, kinetics, and cell fates [153,154,155] (Figure 4A). Haradhvala et al. utilized scRNA-seq and scTCR-seq to identify the expansion and proliferation of memory-like CD8+ CAR-T-cell clones that differentiated into IL7R+ effector memory CAR-T-cells in the tisa-cel responder, while axi-cel responders exhibited more heterogeneous populations [94]. Those CD8+ CAR-T-cells showed a stronger upregulation of activation marker PDCD1 and the immune checkpoint regulator SLAMF6 [94]. 

Even when activated by the same antigen, individual patient’s CAR-T-cell subpopulations displayed different patterns of expansion and different differentiation trajectories [49,94]. In general, clusters with a high expression of cytotoxicity and proliferation genes usually predominated [88,89]. Furthermore, Wilson et al. applied scRNA-seq combined with scTCR-seq methods and discovered an effector precursor CD8+ CAR-T-cell with a unique transcriptional profile, TIGIT^+^CD27^−^CD62^Low^ among the common trajectories of highly effective CD19-specific CAR-T-cells [88]. These CAR-T-cells developed into long-lived memory cells and stayed in the “resting primed” state with minimal energy consumption to prevent relapse [89,156]. For example, Melenhorst et al. reported decade-long leukemia remissions with the persistence of Ki67^hi^CD4^+^ CD4+ CAR-T cells in two patients with CLL [157]. These CD4+ CAR-T-cells harboring a nonclassical memory phenotype characterized by ongoing activation and proliferation expressed cytotoxic genes including Granzyme A (GZMA), Granzyme K (GZMK), and Perforin 1 (PRF1), as well as genes related to oxidative phosphorylation pathways. Those long-persisting CD4+ CAR-T cells exhibited cytotoxic characteristics, proliferation, cytokine expression, metabolic activity, and in vitro response to CAR stimulation, indicating they were functionally active rather than exhausted [157].

A dynamic model of CAR-T-cells changes after infusion has been proposed [156]. Initially, the infusion products are highly metabolically active, with high glycolysis and biosynthetic gene expression; then, they gradually transition to a highly cytotoxic state. Subsequently, the CAR-T-cells become nonproliferative but maintain their cytotoxicity. Eventually, the proliferation and cytotoxicity signatures in the CAR-T-cells decline in the remission phase [156]. Goldberg et al. adapted single-cell analysis combined with mass cytometry simultaneously and discovered that patients’ infusion products showed an upregulation in many trafficking and activation molecules when using leukapheresis T-cells as a baseline. Additionally, they found that CD4 and CD8 trafficking and memory phenotype proteins were significantly enriched in cerebrospinal fluid compared to peripheral blood samples [106]. However, it remains to be elucidated whether it is a CAR-T-cell with memory phenotype that migrates to the CNS or whether the CAR-T-cells that migrate might be reprogrammed once they are in the CNS niche.

More recently, Louie and colleagues utilized a single-cell multiomics analysis and found that CAR+ and CAR-T-cells shared a differentiation trajectory into an NK-like subset after CD19 CAR-T-cell infusion in patients with B-cell malignancies [158].

### 5.2. T-Cell Subset Composition Predicting CAR-T Treatment Failure 

As mentioned earlier, Bai et al. utilized single-cell transcriptomes combined with a surface-protein method to analyze the landscapes of 12 ALL patients and observed that deficient Th2 cell function and excessive differentiation into effector cell phenotypes were associated with CD19-positive relapse [96]. The proteomic data in their study also revealed that a lower frequency of early memory T-cells could predict relapse [96]. 

An increase in regulatory (CAR-Treg) cell populations was observed in axi-cel nonresponders due to the ability to suppress conventional CAR-T-cell expansion and drove late relapses in an in vivo model [78,94]. In addition, Good et al. also identified that a higher level of CD4^+^Helios^+^ CAR-T-cells at day 7 after infusion correlated with clinical progression and milder neurotoxicity by applying single-cell proteomic profiling [78].

More recently, Wang and colleagues utilized single-cell transcriptomes and identified that a larger CD8+ stem cell-like memory T-cell population with prominent activating capacity of the CAR-T-cell infusion products was associated with excellent clinical response. Moreover, they discovered that a loss of CCR7 gene expression, coupled with an increased expression of activation- and inhibitor-related genes in the CD8+ naïve T-cell populations within the apheresis T-cells was associated with poor clinical response. These findings could aid in refining the screening process for T-cells during CAR-T-cell preparation [86]. Li and colleagues utilized single-cell transcriptomes and identified eight CAR-T-cells molecularly subtypes. They further identified that B-featured CAR-T-cells’ subtypes were dysfunctional and associated with a poor clinical outcome [87].

### 5.3. CAR-T Persistence

Deng et al. demonstrated using scRNA-seq in LBCL patients treated with axi-cel, that a complete clinical response was correlated with higher frequencies of CD8+ T-cells expressing memory signatures. In contrast, poor clinical response was associated with a CD8+ T-cell dysfunction signature enriched for exhaustion and activation markers, as well as genes encoding MHC class II proteins [79]. Transcription factors related to CAR-T treatment failure were also highly expressed among poor responders. Nevertheless, patients achieving complete remission had more memory CD8+ T-cells, as indicated by scatter plots of CCR7^+^CD27^+^ CD8+ T cells measured by CapID in their study [79].

### 5.4. CAR-T-Cell Exhaustion

T-cell exhaustion due to prolonged exposure to tumor antigens is considered one of the major causes of immunotherapy failures (Figure 4B). Exhaustion-related transcription factors such as thymocyte selection associated high-mobility group box (TOX), nuclear receptor subfamily 4 group A member 1 (NR4A1), interferon regulatory factor 4 (IRF4) act as central regulators that affect the expression of immune checkpoint genes and drive T-cell exhaustion [63,159,160,161,162]. However, the processes of CAR-T-cell exhaustion differ from the nontransformed T-cells. For example, Deng et al. demonstrated that those DLBCL patients treated with axi-cel with poor clinical response were associated with a CD8+ T-cell dysfunction signature enriched for exhaustion and activation markers, as well as genes encoding MHC class II proteins, using scRNA-seq [79]. Consistently, Singh et al. also demonstrated that treatment resistance was associated with the upregulation of exhaustion markers [95].

Furthermore, scRNA-seq of CAR-T-cells from a first-in-human trial in metastatic prostate cancer demonstrated that the CAR-T-cells of poor responders shifted towards a nonproliferative, highly differentiated, and exhausted state. This group showed that an enriched exhausted profile in CAR-T-cells from patients with an unsatisfactory response was characterized by TIGIT expression [93]. Two transcription factors, BLIMP1 (B lymphocyte-induced maturation protein 1) and NR4A3 (nuclear receptor subfamily 4 group A member 3), also contribute to the regulation of CAR-T-cell dysfunction. A double knockout of these two transcription factors shifted CAR-T-cell phenotypes away from TIM-3^+^CD8^+^ toward TCF1^+^CD8^+^ to counteract the exhaustion of tumor-infiltrating CAR-T-cells, enhancing their antitumor activity in mouse models [163]. In line with this, Jiang et al. used scATAC-seq to demonstrate the landscape of chromatin accessibility of CAR-T-cells during tumor cell stimulation. BATF (basic leucine zipper ATF-like transcription factor) and IRF4 (interferon regulatory factor 4) were significantly enriched in terminally exhausted CAR-T-cells [64].

## 6. Preclinical Stage

### 6.1. On-Target, off-Tumor Toxicity

The selection of target antigens is crucial for CAR-T therapy. However, many target antigens with a universal practical value are often expressed in normal cells, leading to a significant issue known as “on-target, off-tumor” toxicity. To address this challenge, Zhang et al. utilized single-cell analysis datasets of CAR-T target antigens [103]. They developed a comprehensive single-cell atlas for target antigens used in CAR therapy across normal tissues and organs. That study helped identify rare antigen-expressing cell types that might be missed in bulk tissue assessments. Similarly, Jing et al. [104] employed a single-cell approach (CARTSC) to analyze CAR-target gene toxicity at the individual cell level. Their method allowed for the identification of specific targets and the examination of the expression patterns of selected target genes in immune cells and tissues.

### 6.2. Target Antigens and Antigen-Specific Stimulation of CAR-T-Cells

Target antigens and the antigen-specific stimulation of CAR-T-cells play distinctive roles during CAR-T treatment, and the activation mechanisms of CAR-T-cells differ significantly from those of innate T-cells. Several studies have utilized single-cell RNA sequencing (scRNA-seq) and other single-cell sequencing technologies to investigate the heterogeneity of CAR-T-cell products under various conditions, including unstimulated, CAR-induced stimulated, and TCR-induced stimulated CAR-T-cells [75,84,85,101,150].

In detail, under the presence of ligand-independent tonic signaling, unstimulated CAR-T-cells exhibit a combination of early activation, exhaustion signatures, and cytotoxic activities [75]. Upon CAR-induced activation, CAR-T-cells display a highly mixed TH1/TH2 cell signaling profile, and the levels of cytokines, such as IFN-γ, TNF-α, granulocyte-macrophage colony-stimulating factor (GM-CSF), IL-5, and IL-13, show considerable heterogeneity among different cell subsets [109]. Notably, GM-CSF+ CAR-T-cells are considered functionally active due to the high expression of GM-CSF [75,109]. 

Strikingly, both CD4+ and CD8+ CAR-T-cells exhibit a high expression of cytotoxic cytokines, indicating their killing functions [109]. Furthermore, to maintain immune homeostasis after activation, specific CAR-T-cell subsets upregulate the expression of immune checkpoint genes CTLA4 and PD-1, as well as immunosuppressive cytokine genes IL-10 and TGFB1. Simultaneously, they downregulate costimulator genes such as inducible costimulatory (ICOS) and TNF receptor superfamily member 4 (TNFRSF4, OX40) [109]. Interestingly, early signs of exhaustion have been observed in a subset of CAR-T-cells in the initial stages after activation [85,88]. Additionally, gene expression differs between CAR-T-cells stimulated by TCR and those stimulated by CAR, with the former specifically enriched in T-cell activation genes such as IFN-γ, IL-3, and CCL4 [84].

### 6.3. Integration of CAR-Encoding Vectors

The lentiviral vector integration site has been reported to influence clonal expansion. For instance, the integration site within the TET2 gene has been identified as a contributor to clonal expansion [164]. Therefore, the precise localization of the CAR vector in the genome of the T-cell is a crucial factor for successful CAR-T treatment [165,166]. Wang et al. developed a method called EpiVIA for profiling chromatin accessibility and identifying lentiviral integration sites at both the cell population and single-cell levels [107]. Additionally, Charitidis et al. devised a scRNA-seq-based approach to monitor both transduced and untransduced cells during the generation of CAR-T-cells using lentiviral vectors [74].

### 6.4. CAR-T-Cell Manufacturing

Bai et al. employed scRNA-seq in conjunction with CITE-seq to unveil heterogeneities in the transcriptional, phenotypic, functional, and metabolic profiles of donor and patient CAR-T-cells at baseline and following antigen-specific activation [75]. Upon CD19 stimulation, donor CAR-T-cells exhibited a more pronounced activation level, correlated with the upregulation of major histocompatibility complex class II genes, in comparison to patient’s autologous CAR-T-cells [75]. 

Furthermore, the differentiation state and costimulatory domain during manufacturing may also influence CAR-T-cell function. Xhangolli et al. [109] employed high-throughput single-cell transcriptome sequencing, multiplexed single-cell cytokine secretion assays, and live-cell imaging of cytolytic activity to study CAR-T-cells upon antigen-specific stimulation. Both CD4+ and CD8+ CAR-T-cells demonstrated the ability to exert cell-mediated cytotoxicity regardless of the differentiation state. The activation of CAR-engineered T-cells was identified as a primary process leading to a highly mixed response involving both type 1 and type 2 cytokines, along with granulocyte-macrophage colony-stimulating factor [109]. CAR tonic signaling, from CD28z and 4-1BBz CARs, could skew the predominance of particular T-cell subsets within resting CAR-T-cells. The 4-1BBz CARs were enriched in CD8+ central memory cells, in contrast to CD8+ effector and CD4+ central memory cells in the CD28z CAR+ T cell population [84]. Castellanos-Rueda et al. generated a library of 180 unique CAR variants genomically integrated into primary human T-cells using CRISPR-Cas9 [108]. This CAR library offers an integrated approach to CAR-T-cell engineering through signaling domain shuffling and pooled functional screening. scRNA-seq and single-cell CAR sequencing (scCAR-seq) provide high-throughput screening to discover multiple variants with tumor-killing properties and T-cell phenotypes significantly different from standard CARs. These advanced methodologies help expand the combination space of CAR signaling domains and facilitate the potential therapeutic development of novel CAR-Ts [108].

### 6.5. Behaviors and Phenotypes of Individual CAR-T-Cells

Cellular heterogeneity in infusion CAR-T products plays a critical role in the varying efficacy of CAR-T-cell therapy. Understanding the killing behavior and phenotype of individual CAR-T-cells has been a major challenge. LaBelle et al. [167] developed a platform to measure time-dependent CAR-T-cell-mediated cytotoxicity and then isolate single cells for downstream assays. This assay is extremely valuable in the characterization of CAR-T-cells. Given that single-cell resolution accurately captures the degree of similarity between samples, it will be of great importance to study cellular heterogeneity and adequately characterize the interplay among T-cell subsets in CAR-T-cell therapy. 

Xue et al. [71] used a single-cell barcode chip microdevice to demonstrate the diverse landscape of the immune response of CAR-T-cells, providing a new platform for capturing CAR-T product data for correlative analysis. A comprehensive evaluation of preinfusion products paves the way for understanding the relationship between in vitro functional profiles and therapeutic outcomes.

More recently, Wang’s team performed single-cell RNA sequencing (scRNA-seq) to explore the T-cell phenotypes associated with different stages of the production of dual BCMA (B cell maturation antigen)- and TNF receptor superfamily member 13B (TNFRSF13B, TACI) TACI-targeting CAR-T-cells [105]. They demonstrated that tonic signaling occurred in a small proportion of inactivated CAR-T-cells. Additionally, they identified the persistent and distinctive CAR-induced molecular signatures of T-cell activation characterized by high MYC proto-oncogene, bHLH transcription factor (MYC) transcription factor-induced gene expression, limited exhaustion, and a combination with the CD4+ CD8+ effector response. 

Qin et al. [168] designed a type of CAR-T-cells coexpressing chimeric switch receptors specific for PD-L1 and demonstrated that these CAR-T cells could promote differentiation into central memory-like T-cells, upregulate genes related to T helper 1 (Th1) cells, and downregulate Th2-associated cytokines through the CD70–CD27 axis [168]. Importantly, integrative bulk and single-cell profiling of premanufactured T-cell populations revealed that certain factors mediated the long-term persistence of CAR-T-cells. They also performed an RNA-sequencing analysis on sorted T-cell subsets from 71 patients, followed by paired CITE-seq and scATAC-seq on T-cells from 6 of these patients. The study revealed that chronic interferon signaling, regulated by interferon regulator factor 7 (IRF7), was related to poor CAR-T-cell persistence across T-cell subsets. On the contrary, the transcription factor 7 (TCF7) was not only associated with the favorable naïve T-cell state but also with a high number of effector T-cells maintained in patients with long-term CAR-T-cell persistence [168]. More recently, Zhu and colleagues utilized single-cell transcriptomes combined with scATAC and identified FOXP1 and KLF2 as the epigenetic transcriptional checkpoints that regulate the fate and stemness of CD8+CAR-T [91].

Together, single-cell profiling of the behaviors and phenotypes of preinfusion CAR-T-cells provides critical insights into the underlying molecular determinants and could serve as predictors of treatment outcomes (Table 2).

### 6.6. CAR-T-Cell Product Heterogeneity

For CAR structures, the selection of the costimulatory domain is a critical factor to consider. Although CAR-T-cells with CD28 or 4-1BB have similar clinical efficacy, they differ in kinetics and phenotype [9]. Single-cell RNA sequencing (scRNA-seq) has revealed that they exhibit distinct transcriptional expression profiles, both in the baseline and activated states. This divergence may reflect the different transcriptional regulatory mechanisms inherent in these CAR constructs [75,84,169].

It is widely recognized that 4-1BB CAR-T-cells encode the memory phenotype and exhibit longer persistence compared to CD28 CAR-T-cells [84,169]. Studies have indicated that 4-1BB CAR-T-cells express more MHC II genes [75,84,169]. This characteristic is presumed to be beneficial for a coapplication with tumor vaccines to enhance antigen presentation and promote epitope spread. However, it may also elevate the risk of host–graft rejection in allogeneic “off-the-shelf” CAR-T-cells [84].

The difference in manufacturing processes is also crucial and associated with CAR-T-cell product heterogeneity. Previous studies have observed discrepancies in the efficacy of CAR-T-cell products based on the selection of fresh or cryopreserved PBMCs as the primary material [170,171]. A recent single-cell study conducted by Haradhvala et al. suggested that this discrepancy might be related to the decreased number of Treg cells, which are intolerant to freezing [94]. Additionally, the efficiency of the viral transduction of CAR molecules also influences CAR-T-cell fitness and antitumor efficacy. Varied profiles of CAR-T-cells expressed by different CAR molecules (CAR^High^, CAR^Low^) at the bulk and single-cell levels have demonstrated that CAR^High^T-cells exhibit stronger tonic signaling, activation, and exhaustion abilities [172]. Furthermore, the characterization of gene regulatory networks has revealed that CAR^High^ T-cells are regulated by exhaustion-related regulators, including regulatory factor X5 (RFX5), NR4A1, and MAF bZIP transcription factor (MAF) [172]. On the contrary, cells with low or even negative expression of CAR express interferon-induced transmembrane (IFITM) 2 and IFITM3, which prevent viral vector entry and may be potential drug targets to overcome the inefficiency of CAR transduction [74].

In terms of vector bias affecting CAR-T-cell function, single-cell RNA sequencing (scRNA-seq) revealed that the transduction of vesicular stomatitis virus (VSV)-lentiviral vectors (LV) promoted the transition of CAR-T-cells to a central memory phenotype, whereas the transduction of CD8-LV promoted the transition of CAR T-cells to a cytotoxic phenotype [74].

Moreover, CAR-T-cells exhibit significant heterogeneity in cytokine secretion. Polyfunctional CAR-T-cells and the polyfunctionality strength index (PSI) are employed to describe CAR-T-cell subsets capable of coproducing multiple cytokines at the single-cell level. These measures have been utilized to predict the clinical outcome of patients [70,71,72,73].

## 7. Dynamic Interactions between CAR-T-Cells, Tumor Cells, and TME

The tumor microenvironment (TME) plays a crucial role in cancer progression and relapse and is associated with the failure of cancer immunotherapy [173,174]. The function of CAR-T-cells is closely linked to tumor cells, immune cells, and stromal cells within the TME [175]. Moreover, the single-cell analysis of both the tumor and TME helps gain new insights into antigen identification for CAR-T therapy [99,176,177]. Gottschlich et al. utilized an atlas of publicly available RNA sequencing data from over 500,000 single cells and identified CSF1R (colony-stimulating factor 1 receptor) and CD86 (cluster of differentiation 86) as targets for CAR-T-cell therapy in AML [176].

Boulch et al. reported that the interaction between CAR-T-cell subsets and the tumor microenvironment (TME) was essential for sustained cytotoxic activity. Using single-cell RNA sequencing, the authors observed significant changes in the TME during CAR-T-cell therapy [92]. CAR-T-cells did not act independently within the TME but relied on cytokine-mediated crosstalk with the TME for optimal activity [92]. In line with this, Romain et al. [178], using high-throughput single-cell technologies, demonstrated that CD2 on T-cells was related to the directional migration of T-cells. The interaction between CD2 on T-cells and CD58 on lymphoma cells accelerated the serial killing of CAR-T-cells [178]. Additionally, CD4+ CAR-T-cells are more effective in stimulating the host immune response, while CD8+ CAR-T-cells are more powerful in direct tumor cell killing. Both processes require the CAR-T-cell intrinsic expression of IFN-γ. Host sensing of IFN-γ and the production of IL-12 are also necessary for CAR-T-cell function. Therefore, the interaction between CAR-T-cells and the TME is crucial for optimal CAR-T-cell efficacy against tumors [92]. Li X et al., using scRNA-seq on CAR-T-cell products and PBMCs from plasma cell leukemia (PCL), demonstrated extensive interactions between proliferating CAR-T-cells and cytotoxic CAR-T-cells, as well as between CAR-T-cells and endogenous T-cells [156]. The authors showed distinct CAR-T- and endogenous T-cell subsets, indicating stage-specific expression in proliferation, cytotoxicity, and intercellular signaling pathways. Furthermore, we found that CAR-T-cells gradually transitioned to a highly cytotoxic state from a highly proliferative state along a developmental trajectory. Additionally, this suggests that CAR-T-cells may establish a new immune environment by recruiting endogenous T-cells [156].

Indeed, CAR-T-cells serve not only as killers but also as regulators that reshape the tumor microenvironment (TME) and activate both innate and adaptive immunity, producing a synergistic antitumor immunity through the release of IFN-γ [179,180]. Alizadeh et al. reported that IFN-γ derived from CAR-T cells promoted a more activated and less suppressive TME, leading to concurrent activation and an increase in host T lymphocytes and natural killer (NK) cells. This, in turn, upregulated myeloid cells expressing more antigen processing and presentation-related genes, subsequently affecting the killing efficacy of CAR-T-cells in vivo [181]. However, persistent contact between CAR-T-cells and tumor cells leads to CAR-T-cell exhaustion. Good et al. reported that exhausted CD8+ CAR-T-cells transitioned to a NK-like phenotype [100]. Surprisingly, the transcriptional regulators ID3 and SOX4 were specifically expressed in exhausted NK-like CAR-T-cell clusters [168]. These NK-like CAR-T-cell clusters, comprising CD8+ CAR-T cells, acquired NK receptors via plasticity during prolonged antigen exposure [181]. Jiang et al., using scATAC-seq, identified two subsets of exhausted CAR-T-cells. One consisted of intermediate exhausted CAR-T cells with enriched motifs of transcription factors such as Jun proto-oncogene, AP-1 transcription factor subunit (JUN)**,** Fos proto-oncogene, AP-1 transcription factor subunit (FOS), nuclear factor kappa B subunit 1(NFKB1), and BTB domain and CNC homolog 2 (BACH2), and the other consisted of terminal exhausted CAR-T-cells with enriched motifs of BATF, IRF4, and PR/SET domain 1 (PRDM1). In conclusion, the role of IFN-γ is essential for the dynamic interactions between CAR-T-cells and tumor cells in the TME.

## 8. Further Strategies for Future CAR-T-Cell Therapy

### 8.1. Combination Therapy

Combining CAR-T-cells with other therapies such as chemotherapy [182], radiotherapy [183], hematopoietic stem-cell transplantation [184], oncolytic virus therapy [185], small molecules [186], and other immunotherapies has emerged as a promising strategy to further improve the effectiveness of CAR-T-cell therapy [187]. Srivastava et al. [182] employed scRNA-seq to delineate the immune cells of murine lung tumors treated with Ox/Cy and CAR-T-cells, revealing the expression of T-cell-recruiting chemokine genes, including CXCL16 and CCL5, in multiple cell types in the tumor microenvironment (TME), such as macrophages and dendritic cells [170]. Furthermore, IFN-γ produced by tumor-infiltrating CAR-T-cells led to the recruitment and activation of iNOS+ tumor macrophages, further upregulating the expression of the CXCR3 ligands CXCL9 and CXCL10. This binding to CAR-T-cells expressing CXCR3 facilitated a positive feedback loop [182].

In addition, Sen et al. used scRNA-seq technology to investigate the effect of combining CAR-T-cells with stimulator of interferon genes (STING) DMXAA/cGAMP in a breast cancer mouse model. The results showed an increase in proinflammatory myeloid cells, a reduction in myeloid-derived suppressive cells, and an increase in the expression of chemokines that facilitated CAR-T-cell recruitment and persistence at the tumor site [188]. Furthermore, Lelliott et al. applied scRNA-seq and CITE-seq in an ovarian cancer mouse model demonstrating that CDK4/6i might enhance the cytotoxic effects CAR-T-cells, thereby improving the effectiveness and persistence of tumor control [189].

### 8.2. Engineered CAR-T-Cells

Single-cell sequencing technologies play a crucial role in the design of novel engineered CAR-T-cells [190,191,192]. Wang et al., utilizing genome-wide clustered regularly interspaced short palindromic repeats (CRISPR) screening, identified Ikaros family zinc finger protein 2 (IKZF2) and transducin-like enhancer of split 4 (TLE4) as potentially linked with the functional suppression and exhaustion of T-cells in GBM mode. scRNA-seq of CAR-T-cells with a double knockdown of IKZF2 and TLE4 revealed enhanced cytotoxicity and immune stimulation transcriptional signatures while inhibited exhaustion-associated signatures [193]. Zhang et al. and Mueller et al., employing scRNA-seq, found that CAR-T-cells integrating the CAR gene at the T-cell receptor α constant (TRAC) locus and the PD1 gene locus both expressed a memory-like phenotype and fewer exhaustion-associated transcriptional signatures [105,194]. This site-directed integration of CAR genes by CRISPR may be superior to the transitional random insertion by viral vector transduction. Furthermore, Johnson et al. developed a novel CAR-T-cell (RN7SL1 CAR-T cell) that secreted noncoding RNA RN7SL1 in the form of extracellular vesicles (EVs) taken up by immune cells. Researchers performed scRNA-seq on tumor tissues of mice infused with RN7SL1 CAR-T cells, revealing reduced suppressive myeloid cell subsets, increased inflammatory DCs expressing costimulatory genes, and an activated amplification of effector-memory endogenous CD8+ T-cells [195]. Brog et al. developed a novel CAR-T-cell (Super2+IL-33+ CAR-T-cells) overexpressing superkine IL-2 (Super2) and IL-33. They employed scRNA-seq and found that there was a conversion of M2-like macrophages to M1-like macrophages highly expressing antigen presentation genes in the tumor microenvironment (TME) and an upregulation of the ratio between CD8+ effector T-cells and immunosuppressive Tregs in animal models [196].

### 8.3. Locoregional Delivery of CAR-T-Cells

For solid tumors, locoregional and intratumoral CAR-T-cell delivery are also included [28,197]. Intraventricular and intrathecal administration of CAR-T-cells has demonstrated positive outcomes in both preclinical and early clinical trials [22,197,198,199,200,201,202]. Wang et al., applying scRNA-seq and CyTOF, respectively, demonstrated that CAR-T-cells exposed to cerebrospinal fluid (CSF) could promote the formation of a memory-like phenotype through metabolic reprogramming, resulting in a higher antitumor activity [200]. This metabolic reprogramming was accompanied by an increase in the expression of activation markers and trafficking/homing signatures, which may facilitate the migration of CAR-T-cells to the central nervous system [106].

### 8.4. Perspectives for CAR Target Selection

Bosse et al. compared bulk RNA-seq results of neuroblastomas and normal tissues, identifying Glypican 2 (GPC2) as a potential CAR-T-cell target antigen [203].

### 8.5. Challenges

Single-cell sequencing has its limitations and challenges. First, it incurs high sequencing costs, making it suitable for novel discoveries but impractical for large-scale validation. Second, technologically, single-cell sequencing faces several limitations, primarily being applicable to fresh tissue samples [204]. Additionally, sample preparation for certain large tissues, such as bone, poses difficulties. Third, the spatial information of individual cells within the tissue is often lost during isolation. Consequently, researchers should consider incorporating spatial transcriptomics into their studies [205,206]. Lastly, while the most used scRNA-seq technology can uncover novel cell types and their states in heterogeneous tissues, distinguishing immune cells with similar transcriptomes but different functions present a challenge. In conclusion, integrating various types of data is essential, and there is a growing demand for single-cell multiomics technology.

## 9. Conclusions

CAR-T-cell immunotherapy has significantly improved the treatment of hematological malignancies over the past decade. Despite these advancements, challenges such as intrinsic resistance to CAR-T-cell therapy, disease relapse, and serious adverse events persist. Furthermore, the efficacy of CAR-T-cell therapy in solid tumors remains unsatisfactory. Next-generation CAR-T therapy will depend on our evolving understanding of the behavior of engineered T-cells, both in preclinical and clinical settings.

To precisely address the phenotype of individual CAR-T-cells and their dynamics throughout treatment, single-cell sequencing technologies have emerged as crucial tools for research. The analysis of molecular characteristics of individual CAR-T-cells aids in screening for the ideal design of the antigen receptor, preventing lethal off-target effects, guiding gene-based T-cell modification, optimizing CAR-T manufacturing conditions, deciphering the characteristics of CAR-T-cell products, and dissecting the relationships between critical drivers of cancer phenotypes and therapeutic outcomes of CAR-T therapy [207]. Single-cell multiomics technologies serve as valuable tools for research implementation, encompassing single-cell genomics, epigenomics, transcriptomics, proteomics, and spatial transcriptomics. These technologies enable target discovery, the mutual validation of the experimental results, and the exploration of upstream and downstream molecules and pathways.

In summary, single-cell sequencing technologies have illuminated the path for advancing CAR-T-cell therapy in various aspects, spanning processes before CAR-T therapy, the manipulation of CAR-T-cells, the follow-up of CAR-T therapy, and holding the promise to facilitate progress in cancer treatment. By exploring these aspects, we can gain a deeper understanding of CAR-T therapy, ultimately leading to improved clinical outcomes and broader therapeutic applications.

## Figures and Tables

**Table 1:** Overview of FDA-approved CAR-T-cell products.

Product	Target	Viral Vector	Cell Origin before Activation	Leukapheresis Cell Storage	Reference
Tisagenlecleucel (Kymriah)	CD19	Lentivirus	Enriched T-cells	Frozen	[15]
Axicabtagene ciloleucel (Yescarta)	CD19	Retrovirus	PBMCs	Fresh	[16]
Brexucabtagene autoleucel (Tecartus)	CD19	Retrovirus	CD19-depleted and CD4- and CD8-enriched T-cells	Fresh	[17]
Lisocabtagene maraleucel (Breyanzi)	CD19	Lentivirus	CD4 and CD8 T-cells, separately	NA	[18]
Iidecabtagene vicleucel (Abecma)	BCMA	Lentivirus	PBMCs	NA	[32]
Ciltacabtagene autoleucel (Carvykti)	BCMA	Lentivirus	Enriched T-cells	Frozen	[33]

PBMCs: blood; NA: not available.

**Table 2:** Overview of scRNA-seq-based studies on CAR-T-cells.

Technology	Disease	T-cell source	CAR-T Antigen	Findings	Reference
**CAR-T-cell product heterogeneity**					
scRNA-seq, 10x Genomics Chromium Single Cell 3′, Illumina HiSeq bulk RNA-seq	B-ALL	Activated and inactivated CAR-T-cells from 5 healthy donors	LV αCD19–CD28–CD3ζ, LV αCD19–4-1BB–CD3ζ	4-1BB CAR-T-cells have a central memory cell phenotype, enriched expression of fatty acid metabolism genes, and exhibited heightened levels of MHC II genes, ENPP2, and IL-21 axis genes, as well as a reduction in PD1 expression.	[84]
Bulk RNA-seq, CITE-seq, scATAC-Seq 10 × Genomics Illumina Nova-Seq 6000	B-ALL	Postinfusion human CAR-T-cells from 71 patients	LV αCD19–4-1BB–CD3ζ	The TCF7 regulon was not only linked to the favorable naïve T-cell state but was also preserved in effector T-cells in patients exhibiting long-term CAR-T-cell persistence. Chronic IFN signaling regulated by IRF7 was associated with poor CAR-T-cell persistence.	[13]
scRNA-seq, scFTD-seq, Illumina HiSeq 4000 CITE-seq	R/R B-ALL	IPs, activated and inactivated CAR-T-cells of 1 healthy donor and 2 patients	LV αCD19–4-1BB–CD3ζ	Healthy donor-derived CAR-T-cells have stronger functional activities correlated with the upregulation of MHC II genes than patient-derived CAR-T-cells.	[75]
scRNA-seq, flow cytometry	B-ALL	Activated CAR-T-cells of 3 donors	VSV-LV CART and CD8-LV CART	Different lentiviral factors have an impact on CAR-T-cells function. VSV-LV CAR-T-cells produced a more significant central memory phenotype, while CD8-LV CAR-T-cells showed stronger cytotoxic activity.	[74]
scATAC-seq, CHIP-seq flow cytometry	B-ALL, MM	Activated CAR-T-cells, postinfusion CAR-T-cells of 2 patients	LV α CD19 -CD8-4-1BB-CD3ζ BCMA	BATF and IRF4 were key regulators of CAR-T-cell exhaustion.	[64]
scRNA-seq 10 × Genomics Chromium Single Cell 3′; Illumina HiSeq 4000		Autologous CAR T cells from human	(γ-Retroviral) APRIL–CD28–OX40–CD3ζ	Half of the CAR-expressing cells displayed transcriptional changes upon CAR-specific antigen exposure. A small proportion of them displayed exhaustion signatures such as , LAG-3 and TIM-3.	[85]
Bulk RNA-seq, single-cell RNA-seq, and paired T-cell receptor sequencing	DLBCL	Premanufactured source T-cells and CAR-T-cell products	LV α-single-chain bispecific CARs by optimizing the order of anti-CD19 and anti-CD20 scFv’s --CD8-4-1BB-CD3ζ	Loss of CCR7 gene expression, increased expression of activation- and inhibitor-related genes in the premanufactured CD8+ naïve T-cells are associated with a poor molecular response.	[86]
scRNA-seq	B-ALL	Postinfusion human CAR- T-cells from 26 patients	Sequential CD19-22 CAR T. LV α CD19 -CD8-4-1BB-CD3ζ and LV α CD22 -CD8-4-1BB-CD3ζ	Molecularly identified eight CAR-T-cell subtypes. B-featured CAR-T-cells are associated with poor outcome.	[87]
**Dynamic performance of CAR-T-cells**					
scRNA-seq, scTCR-seq 10 × Genomics Illumina NovaSeq	B-ALL	IPs, postinfusion CAR-T-cells of 15 patients	LV α CD19-CD8-4-1BB-CD3ζ	TIGIT^+^CD27^−^CD62L^Low^ is resulting in a highly efficient postinfusion CAR-T-cell phenotype.	[88]
scRNA-seq TCR-seq, 10 × Genomics Chromium Single Cell 50 + V(D)J enrichment, Illumina HiSeq 2500	NHL	IPs, postinfusion CAR-T-cells of 10 patients	LV αCD19- IgG4-CD28-4-1BB-CD3ζ	Clonal diversity of CAR-T-cells was highest in the IPs. Clones expanding after infusion mainly originated from infused clusters with higher expression of cytotoxicity and proliferation genes.	[89]
Single-cell RNA sequencing, protein expression profiling, and T-cell receptor sequencing, as well as mass cytometry analysis	B cell malignancy	Preinfusion and postinfusion circulating immune cells and CAR+ T-cells	donor- derived piggyBac-modified CD19 CAR-T-cells	CAR+ and CAR−T-cells share a differentiation trajectory into an NK-like subset after CD19 CAR-T-cell infusion and associated with good outcome.	[90]
scATAC+ scRNA-seq	B-ALL and melanoma	Mice CD8+ T-cell subsets of CAR-T-cells	RV αCD19-CD28- CD3ζ	Transcription factors functioned as molecular checkpoints of the Tstem-like-to-Teffector -like transition in CD8+ CAR-T-cells. FOXP1 promoted expansion and stemness of CAR-T-cells and limited excessive effector differentiation. KLF2 enhanced effector CD8+ T-cell differentiation and prevented terminal exhaustion.	[91]
**Cellular interactions with CAR-T-cells**					
scRNA-seq 10 × Genomics Illumina HiSeq×10 image analysis, flowcytometry	BCL	BM cells of mice	LV αCD19-CD28- CD3ζ	CAR-T-cells activity depended on cytokine-mediated crosstalk with the tumor microenvironment (TME). IFN-γ produced by CAR-T- cells enhanced endogenous T-cells and sustained CAR-T-cell cytotoxicity.	[92]
**Primary resistance**					
scRNA-seq 10 × Genomics Chromium Single Cell 50 + V(D)J enrichment; Illumina HiSeq 4000	LBCL	IPs from 24 patients	RV αCD19–CD28–CD3ζ (Yescarta)	Exhausted CD8^+^ and CD4^+^ T-cells were significantly enriched in patients with poor clinical response, while memory-type CD8 T-cells were significantly enriched in patients with CR.	[79]
scRNA-seq, 10× Genomics flow cytometry	NHL	IPs, postinfusion CAR-T-cells from 17 patients	LV αCD19-CD8-4-1BB-CD3ζ	Exhausted CD8^+^ CAR-T-cells expressing TIGIT were associated with a poor clinical response in patients	[93]
scRNA-seq, TCR-seq, 10 × Genomics Illumina NovaSeq S4 flow cytometry	BCL	Preinfusion and postinfusion PBMCs, IPs 32 patients	LV αCD19–4-1BB–CD3ζ (Kymriah) (tisa-cel) RV αCD19–CD28–CD3ζ (Yescarta) (axi-cel)	Expansion of proliferative memory-like CD8 clones was a hallmark of tisa-cel response, whereas axi-cel responders displayed more heterogeneous populations. The number of CAR Treg cells was associated with disease progression positively.	[94]
scRNA-seq, 10 × Genomics Chromium Single Cell V(D)J; Illumina HiSeq 2500 genomewide CRISPR/Cas9 knockout screening	B-ALL	IP, postinfusion CAR-T-cells from 2 patients	LV αCD19–4-1BB–CD3ζ	Death receptor signaling was identified as a key regulator of primary resistance to CAR-T-cells in ALL. CAR-T-cells from patients with primary resistance expressed much higher levels of exhaustion markers.	[95]
**Positive relapse**					
scRNA-seq, CITE-seq, Drop Seq Illumina HiSeq 4000 Flow cytometry, multiplexed secretomic assay	ALL	Activated and inactivated CAR T-cells, pre- infusion CAR T-cells from 61 patients	LV αCD19–4-1BB–CD3ζ	The lack of TH2 function in CAR-T-cell products was associated with CD19-positive relapse. Early memory-like T-cell subsets TSCM and TCM were significantly reduced in positive-relapse patients.	[96]
**Negative relapse**					
Bulk RNA-seq, scRNA-seq, flow cytometry	B-ALL	2 second- trimester human fetus HSPCs	CD19 (detail not available)	CD22 preceded CD19 in normal B-cell development and CD34+CD19−CD22+progenitors underlay phenotypic escape after CD19-directed immunotherapies	[97]
**CRS Toxicity**					
scRNA-seq	B-ALL	Post-infusion CD45+immune cells from 8 mice	?V αCD44v6 -CD28–CD3ζ?V αCD19–CD28–CD3ζ	Human circulating monocytes were primarily responsible for the systemic release of IL-6 that caused CRS.	[97]
Single-cell cytokine profiling, flow cytometry	NHL	IPs from 20 patients	RV αCD19–CD28–CD3ζ (Yescarta)	Higher product PSI was associated with clinical and severe CRS. Higher numbers of IL-17A-producing polyfunctional CAR-T (Th17)-cells was associated with severe ICANS.	[73]
**ICANS Toxicity**					
scRNA-seq 10 × Genomics Illumina HiSeq 2500	NA	Human brain, lung pericytes, PBMCs, mice brain cells, mice, and the BRAIN Initiative Cell Census Network public data from GEO	LV αCD19–4-1BB–CD3ζ RV αCD19–CD28–CD3ζ	CD19 was expressed in human brain; mural cells might contribute to the neurotoxicity	[98]
scRNA-seq	BCL	IPs, post infusion CAR-T-cells from 72 patients	LV αCD19–4-1BB–CD3ζ RV αCD19–CD28–CD3ζ	Reactivated HHV-6 carried by CAR-T-cells may enter the CNS. The similar symptoms are similar to ICANS.	[99]
scRNA-seq, scTCR-seq, CITE-seq, CyTOF 10 × Genomics Illumina NovaSeq 6000 or HiSeq 4000	LBCL	Postinfusion CAR-T-cells from 32 patients	RV αCD19–CD28–CD3ζ	CD4^+^Helios^+^ CAR-T-cells on day 7 after infusion manifested hallmark features of Treg cells and were associated with progressive disease and less severe neurotoxicity.	[100]
scRNA-seq	B cell lymphoma	Infusion products of public data from GSE150992		Neurotoxicity is associated with decreasing cycling activity, amount of CAR+ cells, and expression of cell cycle genes and exhaustion-related genes.	[101]
**Hematological toxicity**					
scRNA-seq scTCR-seq 10 × Genomics Illumina NovaSeq	Richter-transformed DLBCL	Pretreatment and post-treatment PB samples	LV αCD19–4-1BB–CD3ζ	Oligoclonal CAR-T-cell expansion as a potential contribution to hematological toxicity.	[102]
**On-target off-tumor effects**					
scRNA-seq, flow cytometry	B-lineage-derived malignant cells, AML and solid tumor-related target antigens	Cells in normal tissues/organs from healthy donors, public scRNA-seq datasets		The expression patterns of 121 target antigens in normal tissues or organs were obtained at the single-cell level.	[103]
scRNA-seq		Cells from the human cell landscape and the adult Human Cell Atlas from 40 donors cell		A more stringent cutoff by defining a CAR target as a potentially risky gene identified targets in public databases that caused potential on-target, off-tumor toxicity.	[104]
**Others**					
scRNA-seq, 10x Genomics Illumina CRISPR/Cas9 genome editing system	B-NHL	IPs, engineered CAR-T-cells , PBMCs from 3 patients	Nonviral AAVS1-αCD19-CD8-4-1BB-CD3ζ	PD1-targeted CAR-T-cells by CRISPR/Cas9 technology and virus-free method.	[105]
CyTOF	B-ALL, NHL, DLBCL	IPs, PMBCs, BM, postinfusion CAR-T-cells from 3 patients	LV αCD19–IgG4-CD28–huEGFRt-CD3ζ	CAR-T-cell product showed increased expression of trafficking and activation molecules, and patients’ CAR-T-cells from peripheral blood, BM, and CSF showed a spatiotemporal alteration in trafficking, activation, maturation, and exhaustion expression, with a distinct signature in the CSF niche.	[106]
scATAC-seq 10 × Genomics Illumina NextSeq 550	BNHL	CAR-T		Developed a method called EpiVIA for the joint profiling of the chromatin accessibility and lentiviral integration site analysis.	[107]
scRNA-seq, scCAR-seq 10 × Genomics Illumina NovaSeq		CAR-T-cells		Generated a library of 180 unique CAR variants genomically integrated into primary human T-cells by CRISPR- Cas9. Identified several variants with tumor-killing properties and T-cell phenotypes markedly different from standard CARs.	[108]
Single-cell, 16-plex cytokine profiling, single-cell barcode chip		CAR-T-cells		Revealed a diverse landscape of immune effector response of CD19 CAR-T-cells to antigen-specific challenge. Significant subsets of stimulated CAR-T cells exhibited a high polyfunctionality with a dominant antitumor effector cytokine profile.	[71]
**Antigen-specific stimulation of CAR-T-cells**					
scRNA-seq, Illumina HiSeq 2500 scFTD-seq; single-cell cytokine assay, single-cell cytotoxicity assay	BCL	Activated and inactivated CAR-T-cells from 3 healthy donors	LV αCD19-CD28-4-1BB- CD3ζ	The activation states of CAR-T-cells exhibited a diverse composition, including TH1, TH2, Treg, and GM-CSF-expressing T-cell subsets.	[109]

Abbreviations: axi-cel, axicabtagene ciloleucel (Yescarta); tisa-cel, tisagenlecleucel (Kymriah); ALL, acute lymphoblastic leukemia; AHCA, adult human cell atlas; AML, acute myeloid leukemia; APRIL, “a proliferation-inducing ligand”, a high-affinity ligand for the receptors BCMA and TACI; B-ALL, B-cell acute lymphoblastic leukemia; BATF, basic leucine zipper ATF-like transcription factor; BBB, blood–brain barrier; BCMA, B-cell maturation antigen; BM, blood marrow; CAR, chimeric antigen receptor; CD86, cluster of differentiation 86; ChIP-seq, chromatin immunoprecipitation sequencing; CITE-seq, cellular indexing of transcriptomes and epitopes by sequencing; CNS, central nervous system; CRS, cytokine release syndrome; CR, complete remission; CRISPR, clustered regularly interspaced short palindromic repeats; CSF1R, colony-stimulating factor 1 receptor; CTLs, cytotoxic T lymphocytes; CyTOF, mass cytometry by time-of-flight; DCs, dendritic cells; ENPP2, ectonucleotide pyrophosphatase/phosphodiesterase 2; FACS, fluorescence-activated cell sorter; FBM, fetal bone marrow; FISH, fluorescence in situ hybridization; GEO, gene expression omnibus; GM-CSF, granulocyte-macrophage colony-stimulating factor; HCL, human cell landscape; HHV, human herpesvirus; hT cells, human T-cells; ICANS, immune effector cell-associated neurotoxicity syndrome; IFN, interferon; IKZF2198, Ikaros family zinc finger protein 2; IPs, infusion products; IRF7, interferon regulatory factor 7; IRF4, interferon regulatory factor 4; LAG-3, lymphocyte activating 3; LBCL, large B-cell lymphoma; LV, lentiviral vector; MCL, mantle cell lymphoma; MHC, major histocompatibility complex; MM, multiple myeloma; NA, not available; NHL, non-Hodgkin’s lymphoma; NK, natural killer; PB, peripheral blood; PBMC, peripheral blood mononuclear cell; PD, progressive disease; PFS, progression-free survival; PR, partial response; PSI, polyfunctional strength index; R/R, relapse and refractory; RV, retroviral vector; scATAC-seq, single-cell assay for transposase-accessible chromatin using sequencing; scFTD-seq, single-cell freeze–thaw lysis directly toward 3’ mRNA sequencing; scRNA-seq, single-cell RNA sequencing; scTCR-seq, single-cell T-cell receptor sequencing; TACI, cyclophilin ligand interactor; TCF7, transcription factor 7; TCM, central memory T-cell; TH, T helper cell; TIGIT, T-cell immunoreceptor with Ig and ITIM domains; TILs, tumor-infiltrating lymphocytes; HAVCR2, hepatitis A virus cellular receptor 2; TLE4, transcription factor transducin-like enhancer of split 4; TME, tumor microenvironment; TRAC, T-cell receptor alpha constant; Treg, regulatory T cell; TSCM, stem cell-like memory T-cell; VSV, vesicular stomatitis virus.

**Table 3:** Overview of scRNA-seq studies on acute lymphoblastic leukemia/lymphoma cells.

Technology	Disease	Cell Source	Target Antigen	Findings	Reference
scRNA-Seq, 10 × Chromium Single Cell 5′	B-ALL	Bone marrow leukemia cells	CD19	CD19-negative leukemic cells were already present prior to CAR-T-cell therapy. This subclone is one of the reasons for relapse.	[113]
scRNA-Seq	B-ALL	CAR-T-cells, leukemic cells	CD19	Surviving leukemic cells cocultured with different conditions (CAR-T-cells and T-cells) for 24 h were analyzed. Leukemic cells with low CD19 expression maintained the reduced CD19 levels through transcriptional programs associated with normal B-cell activation and germinal center reactions. This process facilitated leukemia cell immune escape.	[114]

Abbreviations: ALL, acute lymphoblastic leukemia.
